# Low density lipoprotein - rosiglitazone - chitosan-calcium alginate/nanoparticles inhibition of human tenon's fibroblasts activation and proliferation

**DOI:** 10.18632/oncotarget.21757

**Published:** 2017-10-09

**Authors:** Yi Gong, Jia-Yang Yin, Bo-Ding Tong, Jie-Xi Zeng, Wei Xiong

**Affiliations:** ^1^ Department of Ophthalmology and Eye Research Center, The Second Xiangya Hospital, Central South University, Changsha, Hunan 410011, China; ^2^ Department of Minimal Invasive Surgery, The Second Xiangya Hospital, Central South University, Changsha, Hunan 410011, China

**Keywords:** low density lipoprotein (LDL), rosiglitazone (RSG), human tenon’s fibroblasts (HTFs), glaucoma filtration surgery (GFS), chitosan-calcium alginate - nanoparticles (CSNP)

## Abstract

Anti-fibrotic therapeutic methods with safety and efficiency after glaucoma filtration surgery (GFS) are desirable. In our previous study, by using Human Tenon's Fibroblasts (HTFs) as a model, we proved the expression of peroxisome proliferator activates receptor-γ (PPAR-γ) in HTFs; in addition, rosiglitazone (RSG), an agonist of PPAR-γ, can inhibit transforming growth factorsβ1 (TGF-β1)-induced reactivation of HTFs, thus to inhibit specifically scarring after GFS through intervening TGF-β/Smads signal pathway. However, a better drug delivery way of RSG, to prolong the duration of its function, and to reduce the toxicity of RSG to ocular tissue still remains challenges. Low density lipoprotein receptor (LDLr) is strongly expressed in hyper-proliferation HTFs after GFS. Therefore, we structured targeting LDL-RSG complexes and channel them into HTFs through LDL-LDLr pathway in order to promote anti-proliferation of HTFs and reduce the toxicity to ocular tissue. Meanwhile, in order to improve the release properties of LDL-RSG complexes, we structured slow release system of LDL-RSG/chitosan-calcium alginate - nanoparticles (CSNP), which effectively inhibited TGF-β1-induced HTFs proliferation, synthesis of extracellular matrix and activation of TGF-β1/SMAD pathway. These data suggested that LDL-RSG/CSNP can be a new anti-fibrotic therapeutic method on scarring after GFS and also a novelty administration of RSG.

## INTRODUCTION

Epidemiological data show that, after cataract, glaucoma is second main cause leading to loss of vision in the world, and is the world's second irreversible cause of blindness [1]. At present, the commonly used therapy is glaucoma filtration surgery (GFS), whose effect is mainly affected by the degree of patency of aqueous humor drainage. The postoperative fibrous scar formation is often the main reason for the failure of GFS [2]. Therefore, the regulation of abnormal conjunctival wound repair to prevent the filter scarring has become one of the hot spots in the prevention of glaucoma.

The essence of the scar formation in the filtering tunnel after glaucoma surgery is the local manifestation of the mechanism of wound healing in the artificial filtering area [3]. During this process, the activation of Human Tenon's Fibroblasts (HTFs) has been regarded as the main cause of wound repair and scar formation [4, 5]. Previous studies showed that the HTFs were activated after GFS and transformed into myofibroblasts (MFs) with a characteristic expression of α-smooth muscle actin (α-SMA); the persistence of MFs and the synthesis of extracellular matrix (ECM) such as collagen were important reasons for scar formation after GFS [6, 7]. Transforming growth factors β1 (TGF-β1), an important regulator of wound healing which plays an important role in the formation of scar after GFS, has been regarded as a major inducible factor of HTFs transforming to MFs [8]. In our previous study, we demonstrated that TGF-β1 successfully induced HTF activation [9]. In the present study, TGF-β1-stimulated HTFs were used as cell models to investigate the mechanism and solution strategy of HTFs activation and proliferation.

Rosiglitazone (RSG), a synthetic highly selective agonist of peroxisome proliferator-activated receptor-γ (PPAR-γ), is an insulin sensitizer potent for the treatment of type 2 diabetes mellitus; in recent years, the anti-inflammatory, immune regulatory and anti-fibrosis effects of RSG have also been reported [10–12]. Studies have shown that PPAR-γ agonists play an important role in the regulation of retinal pigment epithelium and corneal fibrosis [13]. In our previous study, we demonstrated that RSG is able to attenuate TGF-β1-induced up-regulation of α-SMA, CTGF, and COL I transcription, as well as affect protein expression, proliferation, and migration of HTFs without toxicity; RSG also can increase PPAR-γ expression and attenuate Smad2/3 phosphorylation under TGF-β1 [9]. Although we have proved that RSG inhibits the scar formation after GFS by interfering with the TGF-β/Smads signaling pathway, a better drug delivery way of RSG, to prolong the duration of its function, and to reduce the toxicity of RSG to ocular tissue still remain challenges.

Low density lipoprotein (LDL) is the major carrier transport of cholesterol in the blood. Due to the proliferating S phase cells needing to absorb a large number of cholesterol for the synthesis of cell membrane [14], the LDL receptor (LDLr) on the cell membrane will be highly expressed. LDLr highly specifically combines with its ligand LDL, and then mediates the endocytosis of LDL. In our previous study, we found that HTFs continue to proliferate upon a variety of stimulating factors; in the meantime, LDLr is strongly expressed on the cell membrane surface of HTFs [9]. These inspired us to combine RSG with LDL to form macromolecular LDL-RSG complex and introduce RSG into the proliferating HTFs through LDL-LDLr pathway.

Recently, polymeric nanoparticles have been a research hotspot in developing novel drug delivery systems [15]. Due to non-toxic and biodegradable characteristics, chitosan/alginate system has been widely studied at the micro-and macro-scales for drug delivery [16]. Numerous researches have confirmed that Chitosan-calcuim-alginate nanoparticles (CNSP) are novel encapsulation agents for drugs as slow release formulations [17, 18, 19–20]. In the present study, in order to verify the effect of RSG on TGF-β1-stimulated HTFs proliferation and fibrosis, and to improve the release properties of LDL-RSG complexes, we structured release system of LDL-RSG/CNSP, which can be a new anti-fibrotic therapeutic method on scarring after GFS and also a novelty administration of RSG.

## RESULTS

### Effects of RSG on TGF-β1-induced HTFs activation and proliferation

**Figure 1:**
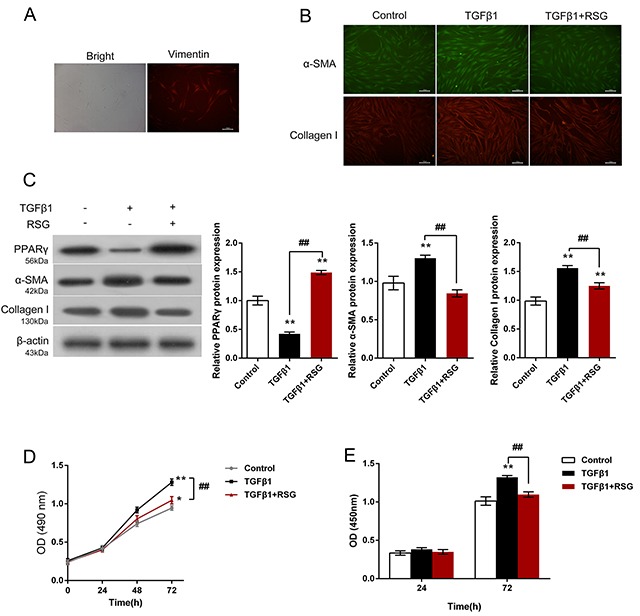
Effects of RSG on TGF-β1-induced HTFs activation and proliferation **(A)** HTFs were isolated and verified by determination of Vimentin. **(B)** HTFs were treated with TGF-β1 or co-treated with RSG and TGF-β1; the contents of α-SMA and Collagen I were determined using IF assays. **(C)** The protein levels of PPAR-γ, α-SMA and Collagen I were determined using Western blot assays. **(D)** and **(E)** The cell viability and DNA synthesis capability was determined using CCK-8 and BrdU assays. The data are presented as mean ± SD of three independent experiments. ^*^P<0.05, ^**^P<0.01, V.S. control group; ^##^*P*<0.01, V.S. TGF-β1 group.

We first obtained HTFs from human Tenon's explants, and then identified the HTFs by determination of cell marker Vimentin (Figure 1A). HTFs were then treated with 5ng/mL TGF-β1 for 48 h or co-treated with 5ng/mL TGF-β1 and 2mg/mL RSG, compared to non-treated control group. Results from IF assays showed that α-SMA and Collagen I content was increased by TGF-β1 treatment, whereas reduced by RSG treatment, compared to control group (Figure 1B). Further, the protein levels of PPAR-γ, α-SMA and Collagen I in HTFs were determined using Western blot assays. Results showed that TGF-β1 treatment significantly reduced PPAR-γ protein level, whereas increased α-SMA and Collagen I protein levels; RSG treatment significantly reversed the effects of TGF-β1 treatment on the indicated protein levels: PPAR-γ protein was increased, whereasα-SMA and Collagen I proteins were reduced, compared to TGF-β1 group (^##^*P*<0.01, Figure 1C). Further, the cell viability and DNA synthesize capability of HTFs were determined using CCK-8 and BrdU assays. Results showed that TGF-β1 stimulation significantly up-regulated HTF proliferation, whereas RSG treatment significantly down-regulated TGF-β1-induced HTF proliferation (Figure 1D and 1E). These data further verified that TGF-β1 induced HTF proliferation and ECM synthesis, whereas RSG significantly reversed the effects of TGF-β1 on HTFs.

### Activation of LDL-LDLr pathway in TGF-β1-stimulated HTFs

**Figure 2:**
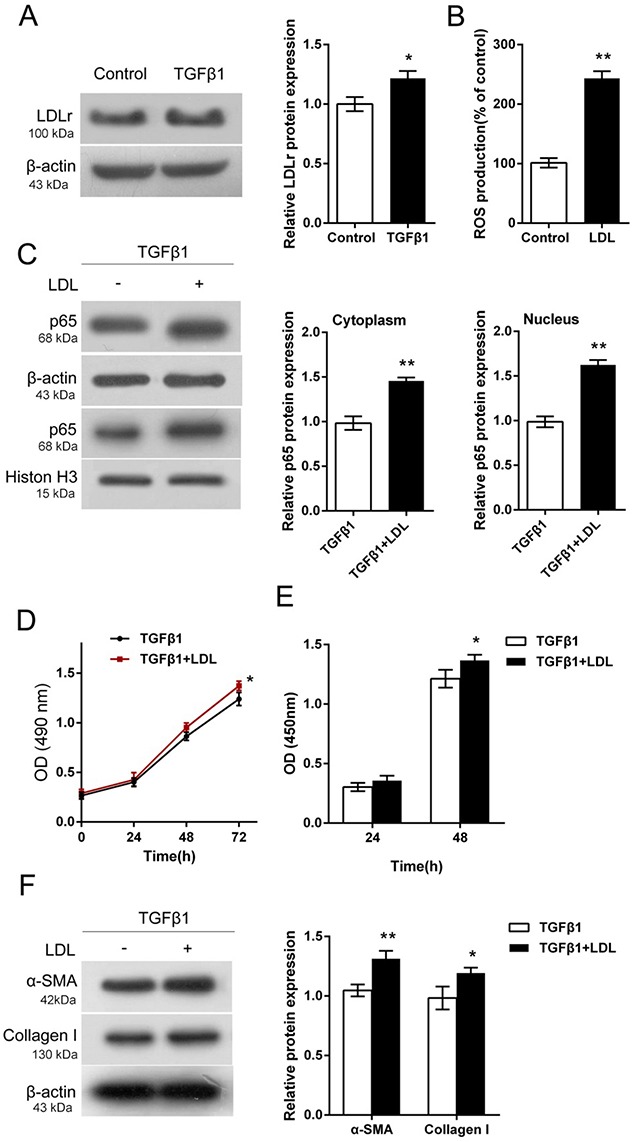
Activation of LDL-LDLr pathway in TGF-β1-stimulated HTFs **(A)** The protein levels of LDLr in TGF-β1-stimulated HTFs were determined using Western blot assays. **(B)** The ROS contents in TGF-β1-stimulated HTFs with the presence or absence of LDL were determined using a DCFH-DA cell-permeant probe. **(C)** The protein levels of p65 in the cytoplasm and nucleus of in TGF-β1-stimulated HTFs with the presence or absence of LDL were determined using Western blot assays. **(D)** and **(E)** The cell viability and DNA synthesis capability of TGF-β1-stimulated HTFs with the presence or absence of LDL was determined using CCK-8 and BrdU assays. **(F)** The protein levels of PPAR-γ, α-SMA and Collagen I in TGF-β1-stimulated HTFs with the presence or absence of LDL were determined using Western blot assays. The data are presented as mean ± SD of three independent experiments. ^*^P<0.05, ^**^P<0.01.

We investigated the function of LDL in proliferating HTFs. HTFs were treated with 5ng/mL TGF-β1, and then the LDLr protein level was determined using Western blot assays. Results showed that TGF-β1 treatment significantly increased LDLr protein level, compared to control group (Figure 2A). Moreover, LDL increased the ROS contents in HTFs compared to control group (Figure 2B). Then, we evaluated the protein levels of p65 in cytoplasm and nucleus upon TGF-β1 stimulation in LDL-treated HTFs. Results showed that p65 protein levels in cytoplasm and nucleus in HTFs were significantly increased by LDL (Figure 2C), suggesting the involvement of p65 in LDL-LDLr pathway activation.

Further, we evaluated the effects of LDL on HTF proliferation. As shown by CCK-8 and BrdU assays, LDL promoted HTF proliferation upon TGF-β1 stimulation, compared to the TGF-β1 group (Figure 2D and 2E). Consistent with the proliferation of HTF, the protein levels of α-SMA and Collagen I were increased by LDL (Figure 2F). These data indicated the activation of LDL-LDLr pathway in TGF-β1-stimulated HTFs.

### Synthesis and evaluation of nanometer microcapsule

**Figure 3:**
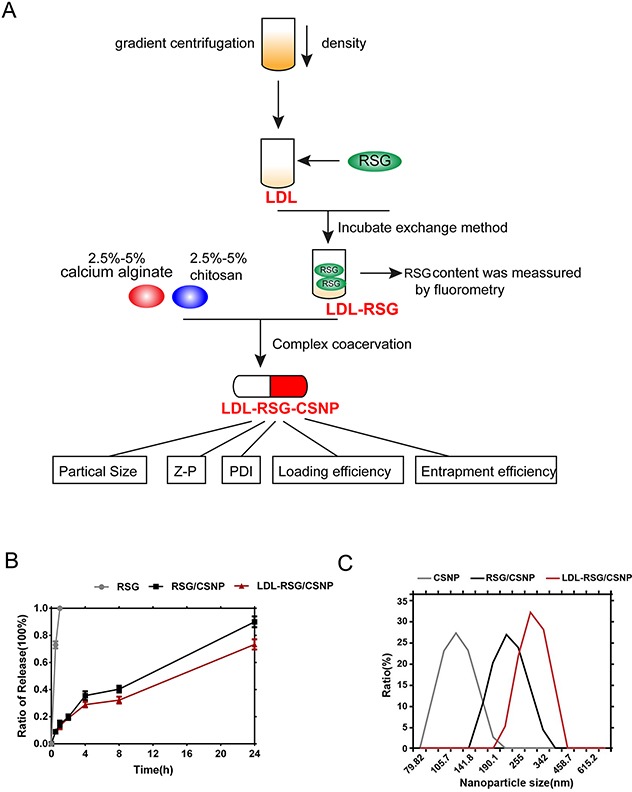
Synthesis and evaluation of nanometer microcapsule **(A)** A schematic diagram showing the synthesis process of LDL-RSG/CSNP. **(B)** The ratio of release of RSG, RSG/CSNP complex and LDL-RSG/CSNP was determined. **(C)** The particle sizes of CSNP, RSG/CSNP complex and LDL-RSG/CSNP was determined using Zetasizer Nano ZS series instrument according to the instruction manual.

After verification of RSG effects and LDL-LDLr pathway activation, we further conducted the synthesis of LDL-RSG-CNSP to figure out a better delivery way of RSG, to prolong the duration of its function, and to reduce the toxicity of RSG to ocular tissue. Figure 3A exhibited the synthesis progression of LDL-RSG/-CNSP. The Average Size, polymey disperse index (PDI), Zeta potential (Z-P), entrapment efficiency (%) and loading efficiency (%) of LDL-RSG/CSNP were showed in Table 1. Then the ratio of release of RSG, RSG/CSNP complex and LDL-RSG/CSNP was determined. As exhibited in Figure 3B, the duration of LDL-RSG/CSNP release was the longest of the three. Further, the particle average sizes of CSNP, RSG/CSNP and LDL-RSG/CSNP were determined using Particle size analyzer (Figure 3C). These results indicated that the LDL-RSG/CSNP was successfully synthesis which showed slow release characteristic.

**Table 1:** The characterization of LDL-RSG-CNSP

Group	Average Size(r.nm)	PDI	Z-P(mv)	Entrapment efficiency (%)	Loading efficiency (%)
LDL-RSG/CSNP	160.1	0.089	11.1	75.65	38.83±2.10
RSG/CSNP	310.2	0.353	28.9	89.23	23.41±1.79
CSNP	354.8	0.32	34.8	N.D	N.D

PDI: polymey disperse index; Z-P: Zeta potential;

### The effects of LDL-RSG/CSNP on TGF-β1-induced HTF proliferation and ECM synthesis

**Figure 4:**
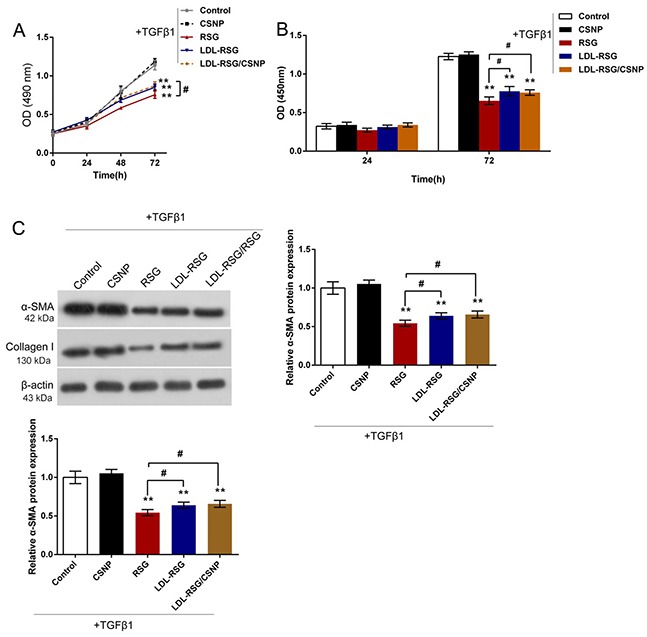
The effects of LDL-RSG/CSNP on TGF-β1-induced HTF proliferation and ECM synthesis **(A)** and **(B)** The cell viability and DNA synthesis capability of TGF-β1-stimulated HTF in response to RSG, LDL-RSG complex and LDL-RSG/CSNP treatment was determined using CCK-8 and BrdU assays. **(C)** The protein levels of α-SMA and Collagen I in TGF-β1-stimulated HTFs in response to RSG, LDL-RSG complex and LDL-RSG/CSNP treatment were determined using Western blot assays. The data are presented as mean ± SD of three independent experiments. ^**^P<0.01, V.S. control and CSNP group; ^#^*P*<0.05, V.S. RSG group.

After successful construction of LDL-RSG/CSNP, we then evaluated the effects of RSG, LDL-RSG complex and LDL-RSG/CSNP on TGF-β1-induced HTF proliferation and ECM synthesis. Results showed that RSG, LDL-RSG complex and LDL-RSG/CSNP could significantly inhibit TGF-β1-induced HTF proliferation (Figure 4A and 4B), as well as the protein levels of α-SMA and Collagen I (Figure 4C), compared to control and CSNP group; although the inhibitory effects of LDL-RSG complex and LDL-RSG/CSNP were slightly weaker than that of RSG (Figure 4A-4C). These data indicated that LDL-RSG/CSNP could efficiently inhibit TGF-β1-induced HTF proliferation and ECM synthesis.

### LDL-RSG/CSNP attenuated activation of TGF-β1/SMAD pathway

**Figure 5:**
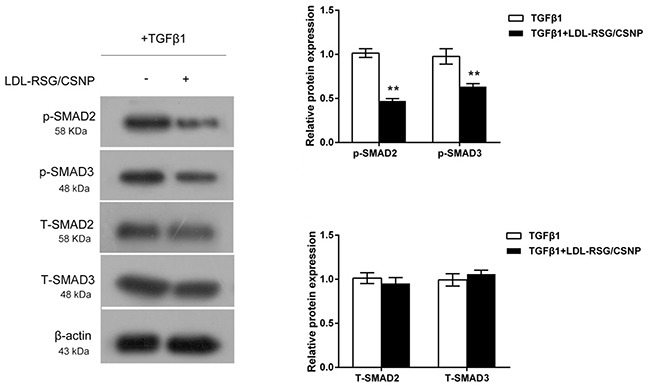
LDL-RSG/CSNP attenuated activation of TGF-β1/SMAD pathway The protein levels of p-SMAD2, p-SMAD3, total SMAD2 and total SMAD3 in TGF-β1-stimulated HTFs with the presence or absence of LDL-RSG/CSNP were determined using Western blot assays. The data are presented as mean ± SD of three independent experiments. ^**^P<0.01.

Our previous study suggested that RSG could suppress the fibrotic effect of TGF-β1 by interfering the phosphorylation of Smad2/3 [9]. In the present study, we evaluated the effects of LDL-RSG/CSNP on activation of TGF-β1/SMAD pathway. Results showed that LDL-RSG/CSNP could significantly reduce the protein levels of p-SMAD2/3 without changes of total SMAD2/3 proteins (Figure 5). These data indicated that LDL-RSG/CSNP obtains a similar inhibitory effect as RSG on Smad2/3 phosphorylation (p-SMAD) induced by TGF-β1.

## DISCUSSION

The transdifferentiation of HTFs to myofibroblasts plays a major role in the wound healing process. In this process, the cells were activated and expressed abundant ECM protein and growth factors that were essential in wound healing [21]. Persistent occurrence of myofibroblast may associate with postoperative scarring and the failure of GFS. Attenuating the transdifferentiation of HTFs to myofibroblasts may improve the long-term effect of GFS. In our previous study, we have demonstrated that RSG, an agonist of PPAR-γ, could efficiently antagonize fibrosis induced by TGF-β1 by interfering TGF-β/SMAD signal pathway *in vitro* [9], thus to prove the scar formation after GFS. In the present study, we obtained HTFs as an expansion culture of the human Tenon's explants, and then verified them by determination of Vimentin. The effects of RSG on cell proliferation and ECM synthesis were then evaluated. Consistent with our previous study, RSG could efficiently inhibit TGF-β1-induced HTF proliferation and ECM protein levels, indicating the potential of RSG being a promising agent of scar treatment after GFS.

However, in addition to the efficiency of RSG, the application of RSG is often associated with severe side effects including weight gain, edema, bone fracture, and congestive heart failure, which have severely limited their clinical application [22, 23]. RSG direct administration can cause all kinds of eye complications. As a key to the treatment of GFS scar, how to extend the duration of the drug in the postoperative operation area, while minimizing its toxic effects on the eye tissue to be resolved. In recent years, many studies have suggested that LDLr levels change in a variety of pathological conditions; LDL-based drug complexes retaining LDL activity can be more easily recognized, picked up and degraded by tumor cells [24]. In our previous study, we revealed a high expression of LDLr on the cell membrane, which make it possible for the LDL-LDLr pathway being a potential molecular target to select LDLr-overexpressed tissues to release the drug. To verify the potential of LDL-LDLr pathway of being the available molecular target, we firstly verified the activation of LDL-LDLr pathway in TGF-β1-stimulated HTFs through determination of ROS contents and cytoplasm and nucleus p65 protein levels. Further, LDL could amplify the effects of TGF-β1 on HTF proliferation and ECM synthesis. In addition, the tendentiousness of LDL combining with lipophilic drugs further strengthens the possibility.

By using the incubation exchange method, LDL-RSG complex was firstly constructed which was subsequently dissolved together with CSNP and magnetically stirred to form LDL-RSG/CSNP. After determination of ratio of release *in vitro* and of particle size, the functions of LDL-RSG complex and LDL-RSG/CSNP on TGF-β1-stimulated HTF proliferation and ECM synthesis were evaluated. Despite the slightly weakness of the inhibitory effects, LDL-RSG complex and LDL-RSG/CSNP could also significantly inhibit TGF-β1-stimulated HTF proliferation and ECM synthesis in HTFs similar as RSG, shown as down-regulated HTF cell viability, DNA synthesis capability and reduced α-SMA and Collagen I protein levels. In the present study, highly expressing LDLr in HTFs together with LDL helps to target the rightful release site of RSG; cationic polyelectrolyte chitosan solution and anionic calcium alginate solution were mixed and stirred to produce CSNP, which is stable, non-toxic and good film-forming [25, 26], thus to steadily and targeted prolong the duration of RSG function in the postoperative operation area with minimum eye tissues toxic.

Further, we investigated the possible downstream signaling through which RSG exerts the functions. In our previous study, we revealed that RSG could suppress the fibrotic effect of TGF-β1 by interfering the phosphorylation of SMAD2/3 [9]. In the present study, we evaluated the effects of LDL-RSG/CSNP on activation of TGF-β1/SMAD pathway. Consistent with our previous study, LDL-RSG/CSNP could significantly reduce the protein levels of p-SMAD2/3 without changes of total SMAD2/3 proteins, indicating that LDL-RSG/CSNP obtains a similar inhibitory effect as RSG on SMAD2/3 phosphorylation (p-SMAD) induced by TGF-β1. However, a clear mechanism between RSG and SMAD2/3 in the gene regulation level is required to be investigated. Some studies had documented that PPAR-γ agonists also have an antiangiogenic activity [27], which was related to its anti-proliferative effects. Some research relating to the antiangiogenic action of this drug is also needed.

In the present study, we structured targeting release system of LDL-RSG/CSNP and channel them into HTFs through LDL-LDLr pathway in order to promote anti-proliferation and anti-ECM synthesis effects of RSG on HTFs and reduce the toxicity to ocular tissue. After construction, the functions and underlying mechanisms were investigated. Taken together, LDL-RSG/CSNP presents a new anti-fibrotic therapeutic method on scarring after GFS and also a novelty administration of RSG.

## MATERIALS AND METHODS

### Human tenon's fibroblasts (HTFs) isolation, culture and stimulation

The protocol complied with the tenets of the Declaration of Helsinki and was approved by the Medicine Human Ethics Committee of the Second Xiangya Hospital of the Centre South University. Tenon's biopsy samples were obtained during standard glaucoma filtering surgery after comprehensive information and written consent of selected patients. HTFs were gained as an expansion culture of the human Tenon's explants and propagated in DMEM supplemented with 10% fetal calf serum (FCS), 100 μ/ml penicillin, and 100 μg/ml streptomycin. HTFs were maintained in the logarithmic growth phase. HTFs at passage 3-6 were used in the study. All experiments were repeated at least three times and averaged values were calculated for analysis in this study.

2mg/mL RSG was prepared and diluted in serum-free DMEM, added to cell cultures medium 1 h before 5ng/ml TGF-β1 stimulation. DMSO (0.1%) was added to the culture medium in the vehicle control wells.

### Immunofluorescence (IF) microscopy

Immunocytochemical analysis was performed by using antibodies against α-SMA (1:300, Cat# 1A4, Abcam) and Collagen I (1:200, ab34710, Abcam) as primary antibodies and FITC-labeled goat against mouse IgG as the secondary antibody. HTFs were grown on coverslips and plated in DMEM with FCS and incubated for 24 h to allow efficient attachment. HTFs were plated in serum-starved medium and pretreated with vehicle or resulting for 2 h. Subsequently the HTFs were incubated with TGF-β1 (5 ng/ml) for 48 h. The HTFs were rinsed in PBS, fixed in 4% paraformaldehyde, permeabilized in triton-100, and blocked in PBS buffer with 5% bovine serum albumin. The cover slips were sequentially incubated with monoclonal α-SMA antibody (1:300) and Collagen I antibody (1:200) as primary antibodies and FITC-labeled goat against mouse IgG as the secondary antibody (1:400) in blocking buffer. HTFs were examined under a laser-scanning microscope (Axio Vert 200, Zeiss, Germany).

### Western blot

After rinsing with ice-cold PBS, HTFs lysates were prepared using lysis buffer (20 mM Tris, 150 mM NaCl, 1 mM EDTA, 1% Triton X-100) containing phosphatase inhibitors (1 mM sodium vanadate, 50 mM NaF) and protease inhibitors (0.1% phenylmethylsulfonyl fluoride; Complete protease inhibitor, Roche). The bicinchoninic acid assay was used to determine protein concentrations. Then, 10 μg of protein extracts were subjected to SDS polyacrylamide gel electrophoresis after being boiled in Laemmli sample buffer. Proteins were transferred to a polyvinylidene difluoride membrane using a Bio-Rad gel-blotting apparatus (BioRad, Hercules, CA, USA). Membranes were blocked in 10% fat-free milk in TBST (10 mM Tris HCl [pH 7.5], 150 mM NaCl, 0.1% Tween20) for 1 h before being incubated with primary antibody (1:1000) overnight at 4°C and with a peroxidase-conjugated secondary antibody (1:5000) for 45 min at room temperature. After each incubation step, membranes were washed in TBST three times (10 min each time). The following antibodies were used: anti-PPAR-γ (ab45036, Abcam, USA), α-SMA (Cat# 1A4, Abcam), anti-Collagen I (ab34710, Abcam), anti-β-actin (Cat# ACTN05 (C4), Abcam), anti-LDLr (Cat# EP1553Y, Abcam), anti-p65 (Cat# E379, Abcam), anti-p-SMAD2 (ab53100, Abcam), anti-p-SMAD3 (ab52903, Abcam); anti-SMAD3 (ab40854, Abcam), anti-SMAD2 (ab40855, Abcam). Peroxidase was revealed by chemiluminescence and visualized by exposure to X-ray films (Kodak, USA).

### Cell counting kit-8 assay

Cell proliferation was checked using cell counting kit-8 assay. Fibroblasts were seeded in 96-well plates with 5,000 cells per well and were treated with the indicated concentrations of vehicle, RSG, and TGF-β1 for 24 h as previous experiments in our study. Then 10 μl of 2-(2-methoxy-4- nitrophenyl)-3-(4-nitrophenyl)-5-(2,4-disulfopheny)-2H-tetrazolium monosodium salt WST-8 was added to each well. The plates were incubated for an additional 4 h at 37°C. Absorbance was measured at 490 nm by using a microplate spectrophotometer and the results were normalized by comparing with the negative control.

### BrdU incorporation assay

By measuring 5-Bromo-2-deoxyUridine (BrdU) incorporation, the DNA synthesis in proliferating cells was determined. BrdU assays were conducted at 24 h and 48 h after HTFs were treated with the indicated concentrations of vehicle, RSG, and TGF-β1 for 24 h as previous experiments. Cells were seeded in 96-well culture plates at a density of 2 × 10^3^ cells/well, cultured for 24 h or 48 h, then incubated with a final concentration of 10 μM BrdU (BD Pharmingen, San Diego, CA, USA) for 2 h. When the incubation period ended, the medium was removed, the cells were fixed for 30 min at RT, incubated with peroxidase-coupled anti-BrdU-antibody (Sigma-Aldrich) for 60 min at RT, washed three times with PBS, incubated with peroxidase substrate (tetramethylbenzidine) for 30 min, and the 450 nm absorbance values were measured for each well. Background BrdU immunofluorescence was determined in cells not exposed to BrdU but stained with the BrdU antibody.

### ROS determination

ROS levels were monitored using a DCFH-DA cell-permeant probe as previously described [28]. Briefly, the cells from different groups were collected and incubated with 10 μmol/L DCFH-DA at 37°C for 20 min and then washed with serum-free medium to remove the extracellular DCFH-DA. The fluorescence was then determined at 488 nm excitation and 525 nm emission using a spectrofluorometer.

### Synthesis and characterization of LDL-RSG-CNSP

Water-soluble chitosan dissolved in ultra-pure water, prepared into 1% (10mg / ml) solution, and then filter with ordinary funnel to remove impurities, stored at 4°C refrigerator. LDL-RSG complex was prepared using the method of incubation exchange 2 mg of RSG was added to a tube containing 2 mg LDL. After shaking, 1 ml of PBS buffer with pH = 7.6 was added to the tube, and shaken at 40°C for 3 h. After keeping in dark 4°C for 48 h, RSG was removed to obtain LDL-RSG complex. Cationic polyelectrolyte chitosan solution and anionic calcium alginate solution were mixed and stirred to produce chitosan-calcium-alginate nanoparticles (CSNP) by intramolecular and intermolecular crosslinking. For LDL-RSG-CNSP, LDL-RSG solution slowly added to the CNSP solution with the magnetic stirring. The characterization of LDL-RSG-CNSP, including The Average Size (r.nm), PDI, Z-P (mv), Entrapment efficiency (%), loading efficiency (%) of LDL-RSG/CSNP, the release time of drug and particle size were measured as previous described [29, 30].

### Statistics

All data of the experiments were presented as means ± SD after calculating the averages of three repetitions. Student's unpaired t test and ANOVA were used to establish statistical significance. Analyses were performed using SPSS 16.0 (Chicago, IL, USA). Statistical significance was accepted at *P*<0.05.
